# The Effect of CacyBP/SIP on the Phosphorylation of ERK1/2 and p38 Kinases in Clear Cell Renal Cell Carcinoma

**DOI:** 10.3390/ijms241210362

**Published:** 2023-06-20

**Authors:** Magdalena Smereczańska, Natalia Domian, Grzegorz Młynarczyk, Irena Kasacka

**Affiliations:** Department of Histology and Cytophysiology, Medical University of Bialystok, Mickiewicza 2C Street, 15-222 Bialystok, Poland

**Keywords:** CacyBP/SIP, ERK1/2, p38, clear cell RCC, renal carcinoma

## Abstract

The prognosis for patients with RCC is very poor because this cancer is diagnosed mainly in the metastatic stage and is resistant to radio- and chemotherapy. According to recent research, CacyBP/SIP exhibits phosphatase activity against MAPK and may be involved in many cellular processes. This function has not been studied in RCC so far, so we decided to test whether CacyBP/SIP has phosphatase function against ERK1/2 and p38 in high-grade clear cell RCC. The research material consisted of fragments of clear cell RCC, whereas the comparative material consisted of the adjacent normal tissues. Immunohistochemistry and qRT-PCR were used to identify the expression of CacyBP/SIP, ERK1/2, and p38. The studies showed an increase in immunoreactivity and gene expression of the parameters examined in clear cell RCC compared with normal tissues. Only in the case of ERK1/2 was it shown that the expression of the *MAPK3* gene was downregulated and the *MAPK1* gene was higher in clear cell RCC. These studies demonstrated that CacyBP/SIP lacked phosphatase function against ERK1/2 and p38 in high-grade clear cell RCC. Further research is needed because a better understanding of the role of CacyBP/SIP and MAPK offers hope for the treatment of urological cancer.

## 1. Introduction

Renal clear cell carcinoma is the most common histopathological subtype of renal cell carcinoma (RCC). Approximately 40% of patients with RCC die from the disease, making it the third deadliest cancer among urological tumors. The risk of developing kidney cancer increases with age, which is confirmed by the fact that the majority of kidney concerns occur in people over 55 years of age (80%). Data demonstrate that the incidence of RCC is much higher in men, who account for almost two-thirds of all cases [1].

CacyBP/SIP is a multidomain and multifunctional protein discovered in the 1990s in mouse Ehrlich ascites tumor cells [2]. CacyBP/SIP, by interacting with various proteins, may affect the processes of proliferation, differentiation, cytoskeleton reorganization, ubiquitination, and stress response [3].

Recent literature data suggest that the role of this protein in various signaling pathways may be related to the dephosphorylation of MAPK. The processes of phosphorylation and dephosphorylation play a key role in most signaling pathways, directly regulating the functions of proteins [3]. The MAPK family includes ERK1/2, JNK, and p38 kinases that regulate the activity of proteins, enzymes, and transcription factors. The range of biological activity of these kinases is very wide, which indicates their participation in the pathogenesis of many diseases [4]. In addition, elevated levels of ERK and p38 kinases were detected in the embryonic rat kidney. These findings suggest that the ERK pathway plays a direct role in the development of renal nephrons, including their size and number, while p38 kinase controls nephron differentiation [5].

Several studies have shown that MAPK can enhance cell proliferation, differentiation, and development in various malignancies as a component of survival signaling pathways [6]. MAPK signaling has been shown to play a role in growth and angiogenesis, and that inhibition of MAPK kinases can disrupt the vasculature, suggesting that MAPK could be used as a therapeutic target for renal cell carcinoma [7]. Furthermore, the MAPK pathway is a critical signaling mechanism involved in the control of glucose uptake by tumor cells. Any abnormal expression or dysregulation of each MAPK component causes cells to develop malignant characteristics. The fact that the MAPK pathway is involved in the development of so many cancers may be the result of its dysregulation due to genetic and epigenetic changes [8].

CacyBP/SIP levels are altered in several cancers, indicating its involvement in maintaining cellular homeostasis. It has been shown that, depending on the type of cancer, CacyBP/SIP may act as a tumor suppressor (e.g., kidney, gastric cancer) or oncogene (e.g., pancreatic, colorectal cancer) [9,10,11]. However, literature reports indicate that reports on kidney, stomach, and breast cancer are ambiguous [12]. Thus, in normal tissues such as the colon or stomach, the CacyBP/SIP protein is almost undetectable, while in colon or gastric tumors, it is highly expressed, and the level of expression is correlated with metastasis [9,13].

According to recent research, CacyBP/SIP exhibits phosphatase activity against ERK1/2 and p38 kinases and may be involved in many cellular processes. So far, only few literature data have shown the ability of CacyBP/SIP to bind MAPK kinases, indicating the participation of this protein in dephosphorylation processes [14]. CacyBP/SIP, by dephosphorylating MAPK kinases, causes significant changes in gene expression, which may result in the participation of this protein in the process of carcinogenesis [15].

Renal cell carcinoma is becoming more common at a rate of about 2.5% per year, and the main reason for this is that the exact molecular mechanisms that cause this cancer are not fully understood. Therefore, it is crucial to increase our knowledge of the mechanisms underlying the most common type of RCC [16].

Preliminary results performed in our laboratory showed that CacyBP/SIP has a phosphatase function against MAPK family kinases in hypertension of various etiologies (unpublished data). This function has not been studied in renal cell carcinoma so far. In this study, we wanted to check whether the CacyBP/SIP protein has a phosphatase function in relation to the ERK1/2 and p38 kinases in human high-grade clear cell renal cell carcinoma.

Given that the main cause of MAPK pathway transduction resulting in tumor progression is mutation in several components of the MAPK pathway, we wanted to investigate the interaction between the CacyBP/SIP and major components of the MAPK pathway.

## 2. Results

A total of 35 patients (20 males and 15 females) with clear cell RCC were evaluated in this study. Normal adherent tissue was also tested in 35 samples.

**Figure 1:**
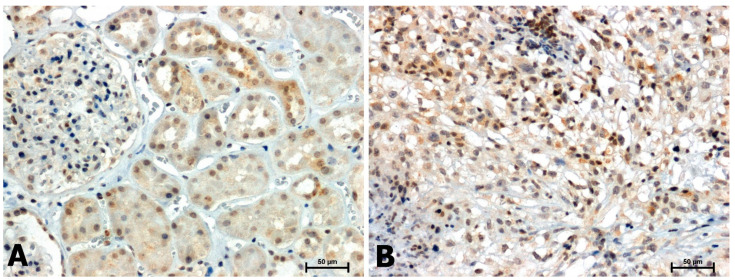
Immunoidentification of CacyBP/SIP in adjacent normal tissues (**A**) and clear cell RCC (**B**). Scale bars: 50 μm.

**Figure 2:**
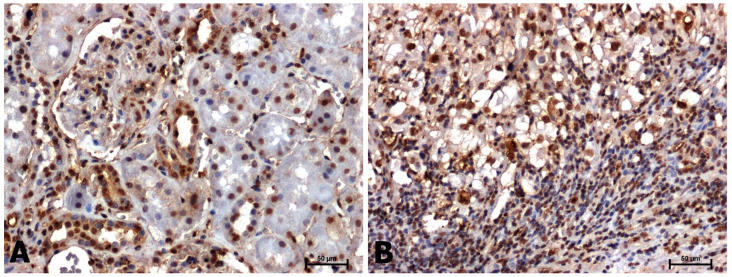
Immunolabeling of p-ERK1/2 in adjacent normal tissues (**A**) and clear cell RCC (**B**). Scale bars: 50 μm.

**Figure 3:**
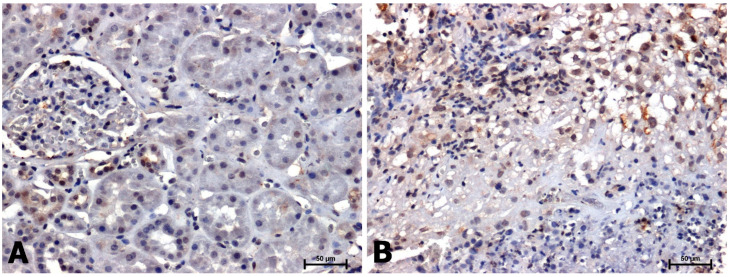
Immunodetection of p-p38 in adjacent normal tissues (**A**) and clear cell RCC (**B**). Scale bars: 50 μm.

A positive immunohistochemical reaction for CacyBP/SIP, p-ERK1/2, and p-p38 was noted in all tissues examined, although the intensity of the immunoreaction was different in non-neoplastic tissues and in samples with clear cell RCC (Figure 1, Figure 2 and Figure 3).

In the normal tissues, moderate to weak CacyBP/SIP immunoreactivity was observed, mainly in the cytoplasm and sometimes in the nuclei of renal tubular epithelial cells (Figure 1A). In contrast, in tumor-bearing tissues, the level of CacyBP/SIP was significantly elevated (Figure 1B).

**Figure 4:**
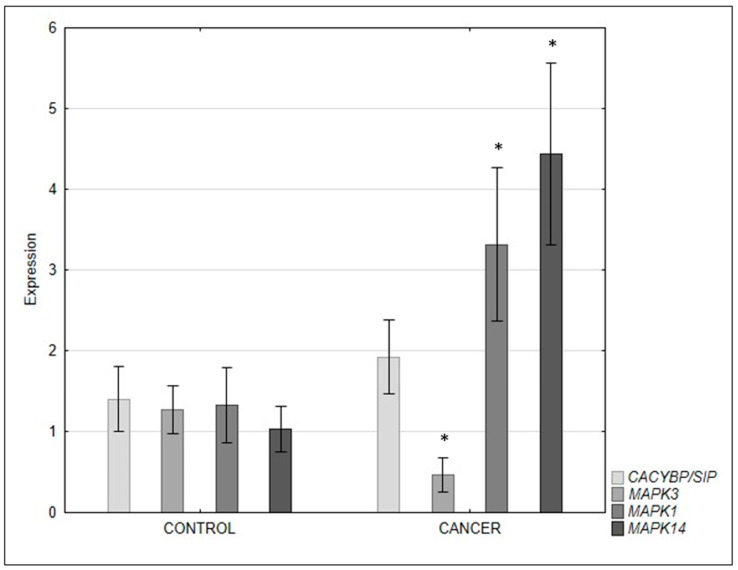
Expression of genes encoding CacyBP/SIP, ERK1/2, and p38 in the normal tissues and clear cell RCC. * p < 0.05—clear cell RCC vs. control.

RT-qPCR analysis confirmed the results of immunohistochemistry, showing an increase in expression of the CacyBP/SIP gene in clear cell RCC compared with adjacent normal tissues; however, this difference was not statistically significant (Figure 4).

Slightly stronger phosphorylated ERK1/2 immunoreactivity was observed in clear cell RCC compared with the level seen in adjacent normal tissue (Figure 2A,B). Immunohistochemical staining showed the presence of ERK1/ERK2 proteins both in the cytoplasm and in the nucleus; usually nuclear localization was dominant (Figure 2A,B).

RT-qPCR analysis showed that the expression of the *MAPK3* gene encoding ERK1 was downregulated and the *MAPK1* gene encoding ERK2 was higher in clear cell RCC than in normal tissues (Figure 4).

In tissues without neoplastic changes, the intensity of the phosphorylated p38 immune signal was very weak in the cytoplasm of epithelial cells of only a few proximal tubules (Figure 3A). Higher p-p38 protein expression, in a similar intracellular location, was observed in sections with clear cell RCC (Figure 3B).

Detailed analysis of RT-qPCR results showed that the expression of the gene encoding p38 in the cancerous kidney was significantly higher than in the adjacent normal tissue (Figure 4).

Computer image analysis confirmed the visually perceived changes in the intensity of the CacyBP/SIP, p-ERK1/2, and p-p38 immunostaining in adjacent normal tissues and in sections with clear cell RCC (Table 1).

## 3. Discussion

Despite the enormous progress that has been made in discovering the pathomechanisms of cancer development, the prognosis in the case of RCC is still poor. Therefore, there is an urgent need to expand knowledge about the molecular mechanisms underlying the development of RCC [16].

A significant part of the search for new agents in the treatment of cancer is related to inhibitors of signal transduction molecules, in particular protein kinases, since many of the genetic changes found in cancer involve genes whose products are signal transduction regulators [17]. Approximately 518 protein kinases make up the human kinome, and more than 150 of these kinases have been implicated in various diseases (http://kinase.com, 28 May 2023). To date, 10 protein kinase inhibitors have been approved, and more than 100 kinase-targeting drugs are being tested in clinical trials [18].

The important role of the CacyBP/SIP protein in maintaining cellular homeostasis has been repeatedly confirmed in both normal and neoplastic tissues. Research studies have shown changes in the level and activity of CacyBP/SIP in several cancers of various organs [14,19].

In the available literature, there are only three reports of CacyBP/SIP expression in kidney cancer with conflicting results; therefore, further research is needed to understand the role of this protein in RCC [9,19,20]. In this study, we demonstrated higher immunoreactivity and increased expression of the CacyBP/SIP gene in clear cell RCC compared with adjacent normal tissues.

Consistent results were obtained in a study comparing three types of renal cell carcinoma: clear cell, papillary, and chromophobe. The authors of this study also showed a significant increase in immunoreactivity and CacyBP/SIP gene expression in clear cell RCC compared with tissues without neoplastic changes. CacyBP/SIP immunoreactivity in papillary and chromophobe RCC was similar to that in the control, but lower compared with clear cell carcinoma [20]. The results of the above studies may indicate that the expression of CacyBP/SIP is different depending on the type of kidney cancer.

On the other hand, Sun et al. [19] demonstrated lower expression of CacyBP/SIP in renal cell carcinoma and renal cell lines A498 and 786-O compared with controls. CacyBP/SIP inhibited the proliferation of kidney cancer cells by affecting the expression of β-catenin. Ectopic expression of CacyBP/SIP in A498 cells delayed cell cycle progression and inhibited cancer proliferation. The same authors showed that injection of CacyBP/SIP mice led to reduced proliferative potential and carcinogenicity in cells overexpressing renal cell carcinoma. Thus, CacyBP/SIP can partially reverse the malignant potential of kidney cancer cells in vitro and in vivo.

Perhaps these conflicting results are due to the fact that Sun et al. [19] do not define the subtype of renal cell carcinoma, while literature data indicate that CacyBP/SIP shows different expression in different types of renal carcinoma [20]. In addition, it should be taken into account that various CacyBP/SIP ligands are involved in carcinogenesis and interactions of CacyBP/SIP with other proteins may inhibit or promote tumor cell proliferation.

Clear cell RCC is considered to have the worst prognosis compared with other types of RCC. A significant correlation was found between the activity of the Wnt/β-catenin pathway and the survival of patients with RCC. Due to the documented participation of CacyBP/SIP in signaling pathways involved in the development of cancer, its connection with the activity of the Wnt/β-catenin pathway cannot be ruled out. Literature data suggest that increased expression of β-catenin in tumor tissue is associated with increased mortality in RCC patients [20]. CacyBP/SIP forms the SCF^TBL1^ complex, recognizing the unphosphorylated form of β-catenin [10]. Since β-catenin plays an important role in processes related to proliferation and tumorigenesis, it can be assumed that CacyBP/SIP regulates the proliferation of cancer cells by affecting the SCF^TBL1^ complex [10,21]. One study showed that CacyBP/SIP, by lowering the level of β-catenin, inhibited the proliferation of kidney cancer [19]. Perhaps the increase in the level of CacyBP/SIP in clear cell RCC, which we have proven, is related to the impaired degradation of CacyBP/SIP ligands, such as β-catenin in cancer cells.

Zhai et al. [9] examined the expression of CacyBP/SIP in many tumor tissues, including 10 renal cell carcinomas, scoring them on a scale of 0 to 4+. Four cases of clear cell RCC were shown to be negative, while in six cases, less than 25% of the cells showed a positive immunohistochemical reaction to CacyBP/SIP.

ERK1/2 and p38 belong to the MAPK family of serine-threonine kinases that regulate proliferation and apoptosis [16]. The best-studied MAPK pathway is ERK, which is dysregulated in one-third of human cancers [22]. Activated ERK1/2 kinase (p-ERK1/2) leads to cell cycle regulation, differentiation, and transformation [15,23]. Chronic inflammation is associated with increased cancer survival. The ability of the p38 pathway to regulate the expression of important inflammatory mediators such as proteases and cytokines that influence tumor growth puts these processes under the control of MAPK pathways [24]. MAPK signaling mutations mainly involve the extracellular signal-regulated kinase pathway, whereas p38 seems to mainly prevent tumor progression. Integration and balance between the above signals are crucial for the compliance and outcome of drug treatment [22].

The present study showed an increase in ERK1/2 immunoreactivity in clear cell RCC; however, the qRT-PCR method showed a decrease in *MAPK3* gene expression and an increase in *MAPK1* expression compared with tissues without neoplastic changes. Perhaps this difference in the expression of the genes encoding the two ERK1/2 isoforms indicates that the *MAPK3* gene is more active in this type of kidney cancer. ERK1 and ERK2 are almost 84% similar and share many functions; however, they are not completely identical. Literature data indicate that the ERK2 subunit has been studied more extensively than ERK1. Both ERK1 and ERK2 are activated by cytokines, osmotic stress, or G protein-coupled receptors; however, the ERK2 subunit leads to dramatic stimulation of phosphatase activity. Literature data have shown that ERK1 and ERK2 are not completely functionally redundant. In addition, it has been suggested that the ERK1 gene is dispensable in mouse development, while ablation of the ERK2 gene is embryonically lethal [25]. An explanation for our results could also be that ERK2 has a proliferative effect and ERK1 an antiproliferative one [26]. Moreover, Sun et al. [26] showed that ERK2 kinase promotes tumorigenesis in renal cell carcinoma. These differences in expression between ERK1 and ERK2 may be of invaluable clinical value due to the possibility of using the determined parameters as potential biomarkers for the early detection of clear cell RCC, as well as a potential target of pharmacotherapy.

In the case of p38, an increase in immunoreactivity and expression of the gene encoding this protein was found.

Huang et al. [7] also revealed higher expression of MAP kinase in human clear cell RCC compared to control tissue. In this study, ERK and p38 phosphorylation decreased after 72 h of LeTx therapy. Suppression of MAPK pathways appears to inhibit RCC cell proliferation by disrupting tumor vasculature.

High expression of ERK1/2 found in cancerogenesis may be the result of strong activation of Ras-Raf-MKK1/2 [15].

Elevated levels of p-p38 have been associated with the malignancy of many cancers, including thyroid, breast, or lung cancer, as well as glioblastoma [24]. Samaras et al. [27] showed that the expression of p38 was correlated with a high Fuhrman’s index in patients with renal cell carcinoma, indicating the usage of p38 suppression as a new therapeutic method in cancerogenesis.

In addition, high-grade RCCs have been shown to have a higher level of MAPK activation than low-grade [28]. This report is consistent with our pilot study of low-grade and high-grade RCC (unpublished data). The results of these studies suggest that the histological grade of cancer may depend on the activation of the MAPK pathway [28].

In addition to the numerous confirmed functions of CacyBP/SIP, recent studies indicate a new phosphatase role of CacyBP/SIP related to MAPK dephosphorylation. CacyBP/SIP interactions with ERK1/2 and p38 are important in the context of the role of these proteins in signaling pathways related to various cellular processes [15]. Literature data indicate that CacyBP/SIP acts as a phosphatase against MAP kinases in cells capable of differentiating, but not in undifferentiated cells [15,23]. The results presented in this paper confirm that CacyBP/SIP lacks phosphatase function against ERK1/2 and p38 in clear cell RCC of G4 grade: undifferentiated (high grade).

Shi et al. [29], in their studies on glioblastoma, also showed that CacyBP/SIP does not exhibit phosphatase activity and promotes the proliferation of cancer cells. Continuing this research, Tang et al. [30] showed that the effect of CacyBP/SIP accumulation in the nucleus and activation of ERK1/2 signaling protects glioblastoma cells against apoptosis.

The lack of CacyBP/SIP phosphatase function can be caused by a number of molecular events. Recent studies have shown that the CacyBP/SIP protein is characterized by two kinase interaction motifs (KIM) that interact with kinases. One is in the C-terminal domain and the other is in the N-terminal domain. Research shows that both motifs are necessary for full CacyBP/SIP phosphatase activity. Perhaps the lack of CacyBP/SIP phosphatase function in clear cell RCC is due to the replacement of lysine at position 25 and arginine at position 26 of the N-terminal KIM motif, resulting in a loss of CacyBP/SIP protein activity as an anti-MAPK phosphatase. In addition, within the same KIM motif there are residues responsible for the formation of the CacyBP/SIP dimer, which indicates a relationship between dimerization and phosphatase activity of this protein [3]. On the other hand, the KIM motif located within the C-terminal domain of CacyBP/SIP coincides with the S100A6 binding site, which explains the competition between ERK1/2 and this protein for binding to CacyBP/SIP. Important factors that may regulate phosphatase activity are also post-translational modifications, in particular phosphorylation, which plays an important role in the regulation the phosphatase activity of the CacyBP/SIP protein [23,31]. An important aspect that may be relevant in our research is the data presented by Wasik et al. [32], who showed that S100A6 and Ca^2+^, by inhibiting CacyBP/SIP phosphorylation on threonine 184, can regulate the activity of CacyBP/SIP as a phosphatase of the ERK1/2-Elk-1signaling pathway.

Several stressors, including the oxidative stress potentially activate MAPK signaling pathways. Reactive oxygen species (ROS) may control cysteine phosphatases and have a direct impact on the cellular pathways. ROS are one of the main causes of carcinogenesis because they play a key role in the processes of metastasis, progression, and apoptosis [16]. Zhong et al. [16] proved that eupatilin, a novel therapeutic agent in the treatment of renal cell carcinoma, promotes ROS-mediated MAPK activation, inhibits the PI3K/AKT cascade, and triggers programmed cell death in RCC cells. As a result, targeting ROS may be a crucial therapeutic approach in cancer treatment.

## 4. Materials & Methods

### 4.1. Sample Collection

The research was carried out on postoperative material collected from 35 patients operated on for kidney cancer at the Department of Urology, Medical University of Bialystok. The study protocol was approved by the Bioethics Committee, of the Medical University of Bialystok (R-I-002/282/2019) and prior written informed consent was obtained from each subject. The research material consisted of fragments of high-grade clear cell RCC lesions obtained during radical or partial nephrectomy. The comparative material consisted of fragments of the surrounding unchanged kidney tissue (margins). Tumorous and normal tissues were immediately fixed in buffered 10% formalin and paraffin embedded in a routine manner or placed in an RNA-later solution (AM7024 Thermo Fischer, Waltham, MA, USA) and stored in −80 °C. Renal paraffin blocks were cut into 4 μm-thick sections and then stained with hematoxylin-eosin for general histological examination and immunohistochemically processed for the detection of CacyBP/SIP, p-ERK1/2, and p-p38. The material stored in the RNA-later solution was subjected to real-time PCR processing to evaluate the expression of the genes encoding CacyBP/SIP, ERK1/2, and p38.

### 4.2. Immunohistochemistry

In the immunohistochemical study, the EnVision method was used according to Herman and Elfont [33]. Immunohistochemistry was performed, using a REAL™ EnVision™ Detection System, Peroxidase/DAB, Rabbit/Mouse detection kit (K5007; Dako Cytomation; Glostrup, Denmark). Immunostaining was performed by the following protocol: paraffin-embedded sections were deparaffinized and hydrated in pure alcohols. For antigen retrieval, the sections were subjected to pretreatment in a pressure chamber heated for 1 min at 21 psi (one pound force per square inch (1 psi) equates to 6.895 kPa; the conversion factor has been provided by the United Kingdom National Physical Laboratory) at 125 °C, using Target Retrieval Solution Citrate pH = 6.0 S 2369 (Dako Cytomation; Glostrup, Denmark) for CacyBP/SIP, p-ERK1/2, and p-p38. After cooling down to room temperature, the sections were incubated with Peroxidase Blocking Reagent S 2001 (Dako Cytomation; Glostrup, Denmark) for 10 min to block endogenous peroxidase activity. Subsequently, sections were incubated with primary antibody for CacyBP/SIP (Rabbit polyclonal to CacyBP ab190950 Abcam, Cambridge, UK), p-ERK1/2 (Rabbit polyclonal to p-ERK1/2, 44-680G Invitrogen, Waltham, MA, USA), and p-p38 (Rabbit polyclonal to p-p38, 44-684G Invitrogen, Waltham, MA, USA). All antibodies were previously diluted in Antibody Diluent Background Reducing (S 3022 Dako Cytomation; Glostrup, Denmark) in relation 1:600 for CacyBP/SIP antibody and 1:50 for p-ERK1/2 and p-p38 antibody. Sections with CacyBP/SIP-, p-ERK1/2-, and p-p38-antibody were incubated overnight at 4 °C (incubation performed in a humidified chamber). The procedure was followed by incubation with secondary antibody (conjugated to horseradish peroxidase-labeled polymer). The bound antibodies were visualized by 1-min incubation with liquid 3,3′-diaminobenzidine substrate chromogen. The sections were finally counterstained in hematoxylin QS (H-3404, Vector Laboratories; Burlingame, CA, USA), mounted, covered, and evaluated under a light microscope. Appropriate washing with Wash Buffer (S 3006 Dako Cytomation; Glostrup, Denmark) was performed between each step. Specificity tests performed for CacyBP/SIP, p-ERK1/2, and p-p38 included a negative control in which the antibodies were replaced by normal rabbit serum (Vector Laboratories; Burlingame, CA, USA) with appropriate dilution. All these controls were negative. Histological preparations were visually analyzed using an Olympus BX43 light microscope (Olympus 114 Corp., Tokyo, Japan) with an Olympus DP12 digital camera (Olympus 114 Corp., Tokyo, Japan) and documented.

### 4.3. Quantitative Analysis

Six sections of malignant lesion and six sections of adjacent normal tissue were examined from each subject (two section for each CacyBP/SIP-, p-ERK1/2, and p-p38-immunostaining). Five randomly selected microscopic fields (each field 0.785 mm^2^, 200× magnification (20× lens and 10× eyepiece)) from each kidney section were documented using an Olympus DP12 microscope camera. Each obtained digital image of the kidney section underwent morphometric evaluation using NIS Elements AR 3.10 Nikon software for microscopic image analysis. The intensity of the immunohistochemical reaction for all the antibodies used in the study was measured on each image analyzed and determined using a gray scale level 0 to 256, where the value of the completely white or bright pixel is 0, while the completely black pixel is 256.

### 4.4. Real-Time PCR

Samples of kidney cancer and non-malignant renal tissue were taken from each patient and placed in an RNA-later solution. Total RNA was isolated using the NucleoSpin^®^ RNA Isolation Kit (Machery-Nagel, Oensingen, Switzerland). Quantification and quality control of total RNA was determined using a spectrophotometer—NanoDrop 2000 (Thermo Scientific, Waltham, MA, USA). An aliquot of 1 μg of total RNA was reverse transcribed into cDNA using iScript™ Advanced cDNA Synthesis Kit for RT-qPCR (BIO-RAD, Hercules, CA, USA). Synthesis of cDNA was performed in a final volume of 20 µL using a Thermal Cycler (Model SureCycler 8800, Aligent Technologies, Santa Clara, CA, USA). For reverse transcription, the mixtures were incubated at 46 °C for 20 min, then heated to 95 °C for 1 min and finally cooled quickly at 4 °C. Quantitative real-time PCR reactions were performed using Stratagene Mx3005P (Aligent Technologies, Santa Clara, CA, USA) with the SsoAdvanced™ Universal SYBER^®^ Green Supermix (BIO-RAD, Hercules, CA, USA). Specific primers for CacyBP/SIP (*CACYBP*), ERK1/2 (*MAPK3*, *MAPK1*), p38 (*MAPK14*), and GAPDH (*GAPDH*) were designed by BIORAD Company (Hercules, CA, USA). The housekeeping gene GAPDH (*GAPDH*) was used as a reference gene for quantification. To determine the amounts of levels of test gene expression, standard curves were constructed for each gene separately with serially diluted PCR products. PCR products were obtained by cDNA amplification using specific primers as follows: *CACYBP* (qHsaCED0043669, BIO-RAD), *MAPK3* (qHsaCID0010939, BIO-RAD), *MAPK1* (qHsaCED0042738, BIO-RAD), *MAPK14* (qHsaCED0043417, BIO-RAD), and *GAPDH* (qHsaCED0038674, BIO-RAD). QRT-PCR was carried out in a doublet in a final volume of 10 µL under the following conditions: 2 min polymerase activation at 95 °C, 5 s denaturation at 95 °C, and 30 s annealing at 60 °C for 40 cycles. PCR reactions were checked, including no-RT-controls, omitting of templates, and melting curve to ensure only one product was amplified. The relative quantification of gene expression was determined by comparing Ct values using the ∆∆Ct method. All results were normalized to *GAPDH*.

### 4.5. Statistical Analysis

All collected data were statistically analyzed by means of software computer package Statistica Version 13.3. The corresponding mean values were computed automatically; significant differences were determined by one-way ANOVA test. For post-hoc analysis, we used the Fisher’s Least Significant Difference (LSD) test; *p* < 0.05 was taken as the level of significance.

## 5. Conclusions

In conclusion, literature data and the results of our own research suggest that the expression of CacyBP/SIP depends on the type and histological grade of renal cancer. In addition, this study provides new insights into CacyBP/SIP phosphatase activity in carcinogenesis. Further studies are needed because understanding what determines the phosphatase function of CacyBP/SIP protein towards MAPK may be of great importance in the future treatment of urological cancer.

## Figures and Tables

**Table 1:** The intensity of immunoreaction determining CacyBP/SIP, p-ERK1/2, and p-p38 in non-malignant tissue and clear cell RCC (mean ± SE).

Intensity of Immunohistochemical Reaction in Kidney Scale from 0 (White Pixel) to 256 (Black Pixel)			
	CacyBP/SIP	p-ERK1/2	p-p38
Control	83.9 ± 0.91	132.7 ± 3.26	76.7 ± 2.83
Clear cell RCC	91.0 ± 2.11 ↑ *	157.4 ± 2.46 ↑ *	103.2 ± 3.41 ↑ *

* *p* < 0.05 clear cell RCC vs. control. **↑**—intensification of immunohistochemical reaction

## Data Availability

Due to patient privacy, no Data Availability Statement was provided for this article.
